# Latest Trends in Investing for Improved Nutrition and Obesity Prevention

**DOI:** 10.1007/s13668-021-00389-7

**Published:** 2022-01-26

**Authors:** Ella Robinson, Rachel Carey, Anita Foerster, Gary Sacks

**Affiliations:** 1 Global Obesity Centre (GLOBE), Institute for Health Transformation, Deakin University, Geelong, VIC Australia; 2 School of Agriculture and Food, The University of Melbourne, Melbourne, VIC Australia; 3 Monash Business School, Monash University, Melbourne, VIC Australia

**Keywords:** Responsible investment, Obesity prevention, Nutrition, Sustainable finance

## Abstract

***Purpose of Review*:**

The aim of this paper is to summarise current trends and new developments with regard to institutional investor actions related to nutrition and obesity prevention.

***Recent Findings*:**

Investor-related activity related to improving population diets is building, with several recent initiatives aiming to accelerate achievement of global nutrition goals. There is increasing civil society and investor activism focused on leveraging investor influence to improve nutrition-related food company actions. There are multiple sustainability-related reporting standards; however, few include comprehensive nutrition-related metrics.

***Summary*:**

There is increasing interest from institutional investors in addressing nutrition-related issues; however, investor activity in the area is piece-meal. There is a need for further integration of nutrition within current reporting frameworks. Methodological alignment across the increasing number of food industry accountability initiatives would likely help galvanise increased investor action. Some jurisdictions are introducing relevant mandatory reporting requirements, which are likely to play a key role in enhancing transparency by the food industry and financial institutions.

**Supplementary Information:**

The online version contains supplementary material available at 10.1007/s13668-021-00389-7.

## Introduction

Over the past 50 years, the way food is produced and consumed has profoundly changed [1]. Population growth, intensive industrial agriculture and food production, combined with neoliberal economic and trade policy arrangements, have led to increasingly globalised and industrialised food systems with wide-ranging negative implications for population and planetary health [1–3]. Comprehensive action from multiple stakeholders is critical for promoting food systems that are healthy, sustainable and equitable [3, 4]. Most food systems are now dominated by the supply, distribution and marketing of processed and packaged foods and beverages [5, 6]. These food system changes have resulted in a shift towards diets dominated by processed, packaged foods that are often low in nutritional value and high in sugar, salt, fat and energy [7]. There is global recognition that the dynamics of modern food systems are a key driver of unhealthy diets and related non-communicable diseases, like obesity, which are the leading contributors to death and disability worldwide [1].

Despite strong calls for widespread government, food industry and civil society action to improve the healthiness of food systems, progress has been limited [3, 5]. Whilst some food companies and retailers have taken steps to address unhealthy diets and obesity, food industry policies and actions have generally been weak and fall far short of global recommendations [8]. Moreover, the increasing market concentration and power of the food industry has meant that key food industry actors (including food and beverage manufacturers, retailers and quick service restaurants) exert considerable influence over governments and policy processes in ways that can either delay or circumvent the implementation of recommended actions to address nutrition issues [9, 10]. Increasing accountability of the food industry for their influence on population diets therefore forms an important component of efforts to prevent obesity and improve health.

The financial sector — including banking, insurance and investment organisations — is increasingly recognizing the importance of incorporating environmental, social and governance (ESG) considerations within financial decision-making (‘sustainable finance’) [11, 12]. There is growing consensus within the sector that this approach can support and even enhance financial growth through risk mitigation and long-term value creation [13, 14]. Within the field of sustainable finance, institutional investors (specialised financial institutions that collect funds from third parties to invest on their behalf in the name of the institution [15]) are particularly well positioned to influence corporate behaviour and governance due to high levels of shareholdings and associated ownership rights (e.g. rights to bring and vote on shareholder resolutions) as well as substantial funds available for new investment [16, 17]. Given that the food industry in many parts of the world has become increasingly consolidated, dominated by shareholder-owned (listed) companies [18, 19] (e.g. large multinational food and beverage manufacturers, retailers and quick service restaurant chains listed on stock exchanges), institutional investors have considerable potential to exert influence in ways that promote increased nutrition-related action and accountability.

The potential role of investors in addressing public health challenges has been reported in academic literature since the early 2000s, most notably in relation to divestment from tobacco [20, 21] and alcohol [22]. Attention to these issues has historically been driven by ethical and faith-based investors, who regard shares in companies that produce these products as ‘sin stocks’ [23, 24]. In regards to health more broadly, a recent study looking at opportunities for scaling up sustainable investment in ‘global health’ by financial markets suggested to add specific ‘health’ (H) criterion to the ESG framework (ESG + H) [25•]. The authors note that this new ESG + H framework could be applied to corporate reporting, responsible investment products and investment decision-making (e.g. screening) [25•]. However, there has been limited academic discussion of investment to address obesity and nutrition challenges.

This paper aimed to summarise the latest global trends and new developments in the approaches taken by institutional investors to nutrition and obesity prevention. We conducted a literature search, predominantly focused on the grey literature. This included relevant reports by leading sustainable financial initiatives (e.g. UN Environment Programme (UNEP) Finance Initiative, European Union Green Deal, Global Sustainable Investment Alliance), global Corporate Social Responsibility and ESG reporting standards, sustainability-related frameworks and conventions. We also conducted a search to identify relevant accountability initiatives, as well as news articles related to investor attention to nutrition.

We firstly provide a brief summary of the rationale and mechanisms for institutional investors to incorporate nutrition-related considerations. We then highlight recent international initiatives, ESG reporting standards and frameworks and accountability initiatives that focus on nutrition-related issues for investors. Finally, we discuss recent trends in investment attention to nutrition and obesity prevention.

## Rationale and Mechanisms for Institutional Investors to Incorporate Nutrition-Related Considerations

**Fig. 1:**
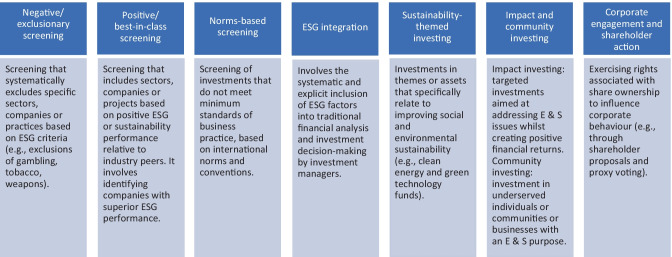
Summary of responsible investment strategies and definitions

‘Responsible investment’ describes investment that considers the ESG performance of companies as well as their financial performance [24]. Responsible investment has its roots in ‘ethical investment’, in which investment decisions were based on ethical or moral values and were primarily faith-based [24, 26]. Today, institutional investor motivations for responsible investment typically also reflect financial goals, including mitigation of financial risks associated with ESG and ensuring sustainable profit growth [24]. Financial regulators and peak finance bodies in many jurisdictions increasingly recognise that ESG considerations are financially material to investment decision-making, and that this is a component of fiduciary duties owed by these institutions to their beneficiaries [14]. By properly taking into account ESG considerations, institutional investors may therefore reduce their exposure to financial risks and potential legal challenges for breach of duty [14]. Institutional investors can integrate ESG considerations within their decision-making through various investment strategies, described in Fig. 1 [27].

The World Economic Forum notes that institutional investors can play a key role in incentivizing the establishment of more equitable, sustainable and healthy food systems through setting higher standards for how companies operating in the food system target environmental and social outcomes alongside financial returns [28•]. Institutional investors that incorporate nutrition within decision-making can mitigate risks related to changing regulatory environments (e.g. taxation and restrictions on sales and marketing of unhealthy products), consumer demand for healthier products and reputational concerns around unhealthy products and unethical business practices [29••]. Given their highly diversified portfolios, institutional investors are likely to be more reliant on a stable and healthy economy (and society) for stronger long-term investment returns [12, 30]. Accordingly, institutional investors may also stand to gain financially from the societal and economic benefits associated with supporting a healthier society through good nutrition.

## International Multi-stakeholder Initiatives that Address Nutrition

With increasing globalisation, the international community has recognised the need for multi-stakeholder governance frameworks that address sustainability challenges, including those related to food systems [31–33]. Many of these initiatives highlight the importance of a comprehensive and coordinated approach to establishing more equitable, sustainable and healthy food systems [31–33]. Below, we highlight some recent international multi-stakeholder initiatives that aim to address nutrition-related issues, with a focus on those relevant to the financial sector.

### The United Nations Sustainable Development Goals

The UN Sustainable Development Goals (SDGs) present a roadmap for society to contribute to the health and wellbeing of people and the planet by 2030 [34]. SDG 2 ‘End hunger, achieve food security and improve nutrition and promote sustainable agriculture’ and SDG 3 ‘Ensure healthy lives and promote wellbeing for all at all ages’ include targets specifically related to the prevention of NCDs and malnutrition^1^ [34]. The 2017 Global Nutrition Report and the World Obesity Federation recognise that addressing malnutrition in all its forms will have wide-ranging impacts on achieving targets within almost all of the SDGs (including through poverty reduction and co-benefits for planetary health) [32, 35].

There are several UN initiatives that aim to mobilise financial sector contributions to achieving the UN SDGs. The UN Global Compact is a voluntary pact for businesses to implement sustainability principles on human rights, labour, environment, anti-corruption and achieving the UN SDGs [36]. The UN Global Compact and KPMG International have developed the SDG Industry Matrix to convert interest stimulated by the SDGs into strategic industry activities which grow in scale and impact [37, 38]. Of note, the SDG Industry Matrix includes suggested actions to support SDG2 (Zero Hunger) and SDG3 (Good Health and Wellbeing) for the Financial Services sector and the Food, Beverage and Consumer Goods sector [37, 38]. The UN Environment Programme (UNEP) Finance Initiative aims to utilize private sector finance to contribute to sustainable development [39]. The UN Principles of Responsible Investment (UNPRI), supported by both the UN Global Compact and the UNEP Finance Initiative, is the leading international network promoting responsible investment, and has over 4000 signatories that have committed to a set of voluntary principles to build a more sustainable global financial system [40]. The UNPRI requires signatories to report on their responsible investment activities in annual transparency reports, including their responsible investment approach for listed equity (where relevant) [41].

### Global Decade for Action on Nutrition

In 2014, the UN and WHO endorsed a global action plan for addressing malnutrition at the ‘Second Conference on Nutrition (ICN2)’ [33]. The ‘Decade of Action on Nutrition’ 2016–2026 provides a directive for UN member states to achieve a broad set of global nutrition and diet-related NCD targets by 2025, as well as the nutrition-related SDGs by 2030 [33]. Of relevance to the financial sector, Action Area 4 of the Decade of Action on Nutrition stipulates the need for trade policy and investment that supports improved nutrition [33]. As part of this action area, responsible investment in agriculture and food systems is nominated as a priority focus [33], although it is unclear what this means in practice for the financial sector. Only three countries — Italy, Ecuador and Brazil — have thus far made SMART (specific, measurable, achievable, relevant and time-bound) commitments to the Decade of Action on Nutrition.

### United Nations Food Systems Summit

In September 2021, the UN convened a Food Systems Summit as part of the Decade of Action on Nutrition to achieve the UN SDGs by 2030 [42]. The Summit was a multi-stakeholder collaboration that aimed to generate action and progress towards achieving all 17 SDGs, whilst raising awareness of how reforming food systems can achieve sustainable development. It is worth noting that the multi-stakeholder event design has been criticized by some for enabling corporate influence [43]. As part of the Summit, ‘finance’ was highlighted as an essential ‘lever of change’ in achieving the Summit aims. The finance community is set to be involved in follow up actions through ‘assessing investment needs, creating incentives, identifying solutions that address inclusion and managing risk’, including by leveraging their resources and mobilizing capital [44].

### Tokyo Nutrition for Growth Summit (N4G)

The Tokyo N4G Summit, scheduled for December 2021, aims to scale up global multi-stakeholder policy action and investment to achieve nutrition-related targets within the SDGs and accelerate the achievement of objectives within the UN Decade of Action on Nutrition [45]. The Tokyo N4G Summit commitments focus on making nutrition integral to Universal Health Coverage for sustainable development; building food systems that promote safe, healthy diets and nutrition, ensure livelihoods of producers and are climate-smart and addressing malnutrition effectively in fragile and conflict-affected contexts. Financing is highlighted as a ‘cross cutting theme’ to achieve these commitments, including the need for ‘innovative financing mechanisms and catalytic funds, and an increased focus on nutrition sensitive financing’ [45].

## Relevant ESG Reporting Standards and Frameworks

There are a multitude of reporting standards and frameworks that investors and other stakeholders use to understand and measure company ESG performance. These include mandatory and voluntary requirements and guidance from regulators, capital markets, professional associations, industry bodies and other organizations [46]. Requirements across jurisdictions remain highly variable, with some jurisdictions moving towards comprehensive and mandatory reporting, and others preferring voluntary provisions [46].

### Governmental-Led Reporting Requirements

Governments can establish legal frameworks, reporting requirements and guidelines for institutional investors and companies in the food industry to contribute towards healthier food systems [28•]. According to the UNPRI, in 2016, 38 of the largest 50 countries (by GDP) worldwide had or were developing some form of government-led corporate reporting requirements for ESG-related issues [47]. We are not aware of any governments that have mandatory corporate or financial sector reporting requirements related to nutrition specifically.

The European Union (EU) is arguably leading the way to improve corporate transparency on ESG issues by mandating ESG reporting requirements as part of the ‘EU Green Deal’ [48]. The EU ‘Corporate Sustainability Reporting Directive’ (2021) requires all large and listed companies (including banks and insurance companies) to report on sustainability-related factors that affect the company, as well as how that company impacts on society and the environment [48]. As well as being mandatory, reporting will be audited in order to bring sustainability information in line with existing requirements for financial information [48]. Additionally, the EU Sustainable Finance Disclosure Regulation will come into force from 2021, and will impose mandatory ESG disclosure obligations for asset managers and other financial markets participants [49].

### Non-Governmental ESG Reporting Standards and Frameworks

There are several key non-governmental ESG reporting standards and frameworks that include relevant nutrition-related topics, and call out financial sector stakeholders as key end users. Two of these include specific nutrition-related reporting metrics for food industry sectors — the sector-specific standards within the Sustainability Accounting Standards Board Standards (SASB) and the GRI Standards (previous G4 Standards and upcoming Sector Standards) [50]. Both provide companies with a framework to report against specific indicators related to topics such as product labelling, marketing, nutritional content and lobbying. The SASB standards include specific, measurable reporting indicators that encourage transparency across 8 food and beverage sectors, including disclosure of revenue from unhealthy products and percentage of marketing impressions made on children [50]. Table 1 provides a summary of nutrition-related topics under relevant reporting standards/frameworks, and Table S1 provides further detail on indicators and metrics under each topic.

**Table 1:** Non-governmental Environmental Social Governance (ESG) reporting standards and frameworks that include relevant ‘social’ or nutrition-related topics^1^

**Organisation**	**Standard/framework**	**Description**	**User**	**Audience**	**Nutrition-related topics**
**The Global Reporting Initiative (GRI)**	GRI Standards [51]	The GRI Standards are one of the most prominent and commonly used sustainability reporting frameworks, providing standards for companies to report on environmental, social and economic impacts. The GRI Standards include a set of universal standards and topic-specific standards that are selected by the company based on materiality	Any organization	Companies, investors, policymakers, capital markets and civil society	GRI 401–419 includes 19 ‘social’ topic specific reporting standards. Standards that may be relevant to nutrition include: • GRI 414: supplier social assessment • GRI 415: public policy • GRI 417: marketing and labelling
	G4 Sector Disclosures (superseded by GRI Standards) [52]	The GRI previously developed the G4 Sector Disclosures, which provided sector-specific reporting guidelines. The G4 Sector Disclosures were superseded by the GRI Standards and are not required for preparing a report in accordance with the GRI Standards, however can still be used to provide additional sector-specific guidance^a^	Airport operators, construction and real estate, electric utilities, event organizers, financial services, **food processing**, media, mining and metals, NGO, oil and gas	Companies, investors, policymakers, capital markets and civil society	Relevant topics under the Food Processing Sector Disclosures include: • **Topic: society** o Public policy o Healthy and affordable food • **Topic: product responsibility** o Customer health and safety o Product and service labelling o Marketing communications
**Sustainability Accounting Standards Board (SASB)**^**b**^	**SASB Standards **[50]	The Sustainability Accounting Standards Board (SASB) provides sustainability accounting standards for companies to disclose financially material environmental, social and governance information to investors. Includes qualitative and quantitative metrics to measure performance across ESG topics	Consumer goods, extractives and minerals processing, financials, **food and beverage**^**c**^, health care, infrastructure, renewable resources and alternative energy, resource transformation, services, technology and communications, transportation	Investors	Relevant topics for restaurants: • **Topic: nutritional content** o Meal options/children’s meal options consistent with national dietary guidelines o Advertising impressions made on children o Promotion of products that meet dietary guidelines for children Relevant topics for processed foods, non-alcoholic beverages: • **Topic: health and nutrition** o Revenue from low/no calorie beverages *(Non-alcoholic beverages only)* o Revenue from products labelled and/or marketed to promote health and nutrition *(processed foods only)* o Process to identify and manage products/ingredients related to nutritional and health concerns • **Topic: product labelling and marketing** o Advertising impressions made on children o Non-compliance with labelling and marketing codes o Monetary losses (due to legal proceedings) associated with labelling and/or marketing practices o Revenue from products labelled as genetically modified organisms (GMO)/non-GMO Relevant topics for food retailers and distributors • **Topic: product health and nutrition** o Revenue from products labelled and/or marketed to promote health and nutrition o Process to identify and manage products/ingredients related to nutritional and health concerns • **Product labelling and marketing** o Non-compliance with labelling and marketing codes o Monetary losses (due to legal proceedings) associated with labelling and/or marketing practices o Revenue from products labelled as genetically modified organisms (GMO)/non-GMO
**Global Reporting Initiative (GRI) and United Nations Global Compact**	**Business Reporting on the Sustainable Development Goals (SDGs)**	Business reporting on the SDGs is an initiative aimed at companies that leverages the GRI Standards and the Ten Principles of the UN Global Compact to embed the SDGs within existing business and reporting processes. The initiative publishes guidelines for companies to integrate SDGs within corporate reporting	All businesses, regardless of size, sector or operating location	Shareholders and other stakeholders: governments, civil society, consumers and academia	SDG 2 Zero Hunger **Target 2.1: Possible relevant business actions** • Respecting access to safe and nutritious food • Recognizing businesses’ own influence on hunger and people’s access to food • Educating the public on the principles of nutrition • Improving the availability of nutritious food Target 2.2: Possible relevant business actions* • Recognizing businesses’ significant influence on people’s diets and access to food • Providing food that contributes to a healthy and balanced diet • Pricing nutritious food options fairly to enable people to afford it considering their purchasing power • Providing sufficient information about products, including nutrition information • Raising the awareness of employees on health issues SDG 3 Good health and wellbeing Target 3.3: Possible relevant business actions* • Encouraging healthy lifestyles • Providing decent working conditions, such as access to affordable nutritious food for mothers in the workplace • Combatting disease and malnutrition by providing adequate nutritious foods and clean drinking-water Target 3.4: Possible relevant business actions* • Supporting access to preventative health care • Taking responsibility to protect consumers and end-users from any potentially negative health impacts from ingredients, products, services and marketing activities • Supporting governmental efforts to reduce non-communicable diseases
**Climate Disclosure Standards Board (CDSP)**	**CDSB Framework** **for reporting** **environmental and** **climate change** **information **[53]	The CDSP offers a framework for corporate reporting of environmental information in mainstream reports, which aligns with and complements the objective of financial reporting (using metrics and KPIs developed by other standards organizations like CDP, GRI, SASB). It allows investors to assess the relationship between specific environmental matters and the organization's strategy, performance and prospects	Organisations, including single companies or entities and corporate groups	Investors	The CDSP is working to expand the scope of its reporting framework to include financially material social issues, with the aim of releasing the new framework in 2021 [54]. It is not clear whetherhealth or nutrition will be included within the scope of this new framework
**The International Integrated Reporting Council (IIRC)**^**b**^	**International Integrated Reporting (< IR >) Framework **[55]	The purpose of the < IR > Framework is to establish principles and elements of an integrated report.An integrated report is a concise communication about how an organization’s strategy, governance, performance and prospects, lead to the creation, preservation or erosion of value over the short, medium and long term	Private sector, for-profit companies of any size. Can also be applied, adapted as necessary, by public sector and not-for-profit organizations	Providers of financial capital	The IR Framework defines various forms of capital (resources and relationships used and affected by the organization), including financial, manufactured, intellectual, human, **social and relationship**, and natural. Company activities related to nutrition may fit under social and relationship capital, through impacts on stakeholder relationships, intangibles associated with brand reputation and the organization’s social license to operate

^1^E﻿SG reporting standards and frameworks that only include topics related to environment/climate change are not included in this table. The most prominent of these are the Taskforce on Climate related Financial Disclosures (TCFD) and the Climate Disclosure Project (CDP). Only nutrition and nutrition-related non-communicable disease relevant business actions/opportunities were extracted and reported. Table 1 includes a summarized version, a full list of nutrition-related business actions/opportunities can be found in Table S1.

^a^The GRI is working on new ‘Sector Standards’ which will describe a sector’s most significant impacts from a sustainable development perspective. As at 2021, standards have been developed for the ‘Oil and Gas Sector’ and ‘Agriculture, Aquaculture and Fishing Sector’.

^b^In November 2020, the International Integrated Reporting Council (IIRC) and the Sustainability Accounting Standards Board (SASB) announced their intention to merge into the Value Reporting Foundation, which was officially formed in June 2021. The SASB Standards are now maintained under the auspices of the Value Reporting Foundation.

^c^Food and beverage includes 8 industries: food retailers and distributers, meat, poultry and dairy, alcoholic beverages, non-alcoholic beverages, processed foods, restaurants, tobacco and agricultural products.

More broadly, the SDGs have been used as a reporting framework for nutrition-related issues. For example, the GRI and the UN Global Compact ‘Business Reporting on the SDGs’ initiative outlines company actions that have the potential to support the SDGs [56]. For SDG2 (Zero Hunger) and SDG3 (Good Health and Wellbeing), these include, for example, actions related to improving the availability of nutritious food and protecting consumers from negative health impacts associated with products and marketing activities (see further details in Table 1 and Table S1). However, a 2020 survey of sustainability reporting found that corporate reporting on the SDGs almost exclusively focuses on positive contributions towards the goals, and lacks transparency with regard to negative impacts [57]. Moreover, another study found that, whilst the SDGs are a major focus for investors and companies, few reporting requirements and resources provide for business disclosures on SDG2 and SDG3 [46]. One study suggested that for listed companies, investment opportunities for SDG3 are seen as ‘high’, but the focus is primarily on the healthcare sector, rather than nutrition. For SDG2, whilst the authors identify a ‘role’ for nutrition companies, the extent and types of investment opportunities that can be accessed through listed companies are estimated as ‘low’ overall [58].

## Benchmarking and Accountability Initiatives

In the past 5 years, there has been substantial growth in the number of food industry benchmarking and accountability initiatives led by civil society organizations. These initiatives are useful for investors, who can use nutrition-related corporate performance data in their research, engagement and analysis, whilst identifying leaders and laggards [59, 60]. Table 2 provides details on food industry benchmarking and accountability initiatives that include various aspects of nutrition as a key focus area.

**Table 2:** Food industry benchmarking and accountability initiatives that include nutrition as a key focus area

**Initiative**	**Industries included**	**Country/jurisdiction**	**Topics covered**	**Details**
**Access to Nutrition Initiative (ATNI)**	Food and beverage manufacturers	Global indices; spotlight indices in India, the USA	Labelling, marketing, governance, accessibility, lifestyles, engagement; product profile assessment; breast milk substitutes and complimentary foods	ATNI benchmarks major global food and beverage manufacturers on their nutrition and undernutrition related policies and practices and assesses the healthiness of their product portfolios. ATNI also conducts in country assessment of food company breast milk substitute (BMS) and complimentary foods (CF) marketing practices and policies. The results of the BMS assessment are also used by the FTSE4Good Index* The ATNI publishes its flagship global index every 2–3 years, the most recent being 2021 [61], as well as spotlighting indices in India [62] and the USA [63]. ATNI has a strong focus on investor engagement, and at least 74 investment organisations representing over US$16.5 trillion assets under management have signed on to the ATNI Investor Expectations on Diets, Nutrition and Health [59, 64]
**ShareAction (in conjunction with ATNI)**	Food retailers	UK	Governance, nutrient profiling, product formulation, in-store promotion, responsible marketing, labelling, engagement, infant and young child nutrition	ShareAction, a UK-based charity, leads a Healthy Markets Initiative aimed at leveraging institutional investment to improve the obesity and nutrition-related performance of food retailers and manufacturers in the UK [57]. ShareAction has a focus on shareholder advocacy and building investor coalitions to engage with food companies on nutrition and health issues. This includes improving policies and practices related to the healthiness and affordability of products, advertising of sugary products to children and food labelling. In 2020, the Access to Nutrition Initiative in collaboration with ShareAction benchmarked the 10 largest food retailers in the UK (ALDI UK, Asda, Co-op, Iceland, Lidl, Marks & Spencer, Morrisons, Sainsbury’s, Tesco and Waitrose) on their nutrition policies and commitments [65]
**World Benchmarking Alliance (WBA)**	Agricultural inputs, agricultural products and commodities, animal proteins, food and beverage manufacturers/processors, food retailers, restaurants and food service	Global	Social inclusion, governance and strategy, environment, nutrition	WBA (a non-profit organisation that assesses influential companies on their contribution to the SDGs) has developed the Food and Agricultural Benchmark to assess 350 of the world’s largest food and agricultural companies on their commitments across three key domains of food systems: environment, social inclusion and nutrition [66]. Companies assessed in the 2021 benchmark include agricultural inputs, agricultural products and commodities, animal proteins, food and beverage manufacturers/processors, food retailers, restaurants and food service [67]
**Plating Up Progress**	Supermarkets, quick service restaurants, contract caterers and food service, casual dining and restaurants and wholesalers	UK	Nutritious products and services, encouraging healthy diets, climate change, biodiversity, sustainable food production, water, food waste and loss, plastics, animal welfare and antibiotics, human rights	Plating Up Progress is an initiative by the Food Foundation, a registered charity in the UK, to assess and measure food industry performance across 10 major topics related to healthy, sustainable and just food systems. The intended end users are investors, businesses and governments. In 2020 and 2021, Plating Up Progress assessed the commitments, targets and performance reporting of major UK supermarkets and caterers, quick service and casual dining restaurant chains [68]. Plating Up Progress is also working with the WBA and others to align metrics for assessing the food industry on their performance in relation to healthy and environmentally sustainable food systems and has a significant focus on useful metrics for investors [60••, 69]
**INFORMAS** (the International Network for Food and Obesity/NCDs Research, Monitoring and Action Support)	Food retailers, quick service restaurants, food and beverage manufacturers	Australia, New Zealand, Canada, Malaysia, Thailand, Europe	Product formulation, nutrition labelling, corporate strategy, relationships with external groups, promotion practices, product accessibility	INFORMAS is a global network of public‐interest organisations and researchers that aims to monitor and benchmark food environments, active in more than 60 countries [70, 71]. As part of INFORMAS, the Business Impact Assessment — Obesity and population nutrition (BIA-Obesity) is an initiative that benchmarks the nutrition‐related policies, commitments and practices of food and beverage companies, food retailers and quick service restaurants [72]. The BIA-Obesity was developed based on ATNI methods [72]. The assessment of food industry policies and commitments as part of BIA-Obesity has been completed in several countries/jurisdictions (Australia [73], New Zealand [74], Canada [75], Malaysia [76], Belgium [77] and parts of Europe [78])

*The FTSE4Good Index Series is designed to measure the performance of companies demonstrating strong ESG practices. An important component of the criteria used to assess corporate practices and performance is the Breast Milk Substitute (BMS) Marketing Criteria. These form part of the Customer Responsibility Theme in FTSE Russell’s ESG Ratings methodology and form a requirement threshold for inclusion in the FTSE4Good Index Series. Companies which manufacture BMS products must meet these BMS Marketing Criteria in full in order to enter the index series [79]

## Investor Attention to Obesity and Nutrition-Related Issues

There is limited academic research exploring the extent to which obesity and nutrition-related issues are considered by the financial sector. A recent study looking at the commitments of 35 leading responsible asset management companies and superannuation funds in Australia found 18 out of 35 investors reported incorporating nutrition-related considerations within their decision-making, albeit in limited ways. Examples included investors actively engaging with food companies to encourage improved nutrition-related policies and practices, and screening food companies based on the healthiness of their product portfolios [80••]. Another previous study reported that whilst obesity-related issues are incorporated within the disclosure requirements and assessment indicators for widely used ESG reporting initiatives, such as the GRI, they made up only a minor component of such initiatives [81].

In the grey literature, several asset management and ESG data analytics companies have reported on obesity and nutrition as a financially material issue facing the food and beverage industry. Shareholder advocacy and engagement around nutrition issues is also emerging. Several recent examples are summarized below.

### Investor Research and Reporting on Nutrition and Obesity-Related Issues

In 2017, Schroders and Rathbone Greenbank Investments (both asset management firms) released a report on the risks surrounding sugar, obesity and NCDs and their expectations for companies that are exposed to unhealthy products (particularly sugar) [82••]. The report noted that litigation, regulation and consumer preference changes all pose risks to the earnings of the food industry, and estimated the potential impact on earnings per share to be 3–25%, depending on the company’s ‘exposure’ to sugar-related risks. Of note, they outline specific investor expectations for companies where sugar/unhealthy products are material risks across 5 core areas.^2^ In a similar vein, a 2013 report by Credit Suisse, a global investment bank and financial services company, discussed trends in sugar as an ESG issue [83]. This report highlighted the risks facing the food and beverage industry due to a surge in negative public opinion surrounding sugar and threats of regulation and taxation. Credit Suisse noted that the extent to which companies can manage risks related to sugar without hurting their current business models was unclear; however, the report did not outline specific expectations for companies with significant exposure to sugar.

Outside of sugar and unhealthy products-related issues, investor interest in the rapidly growing alternative proteins market (estimated to reach $290 billion by 2030) has substantially increased over the past several years, particularly from venture capitalists [84]. A 2021 report by BCG, a management consulting firm, noted that alternative proteins (including meat and dairy alternatives and cell-cultured meats) represent attractive investment options and a tangible way for investors to address ESG concerns [84]. However, the framing around alternative proteins is primarily related to environmental and ethical benefits, with little attention to nutrition. From a public health perspective, several concerns have been raised around the nutritional profile and ultra-processed nature of these products, as well as risks that heavily marketed alternative proteins may replace whole foods plant-based proteins in the diet [85–87].

### Recent Examples of Shareholder Advocacy and Engagement on Nutrition and Obesity-Related Issues

Shareholder advocacy and engagement on nutrition issues is building, particularly in the UK where groups like ShareAction and the Food Foundation are particularly active. In 2021, Rathbone Greenbank Investments led a coalition of investors representing £2.8 trillion in assets (alongside ShareAction and the Food Foundation), urging the UK government to demonstrate leadership and ambition in its response to the National Food Strategy’s recommendations for promoting a healthy and sustainable food system [88]. Of note, the investor coalition strongly supported calls to mandate food company reporting on their products and sales [88].

Also in 2021, an investor coalition coordinated by ShareAction filed a shareholder resolution at Tesco asking the major UK retailer to disclose the share of total food and beverage product sales by volume made up of healthier products, develop a strategy to increase that share by 2030 and publicly report on progress [89•]. ShareAction reported that in response to this resolution, Tesco committed to increase the proportion of sales from healthier products (including a specific time bound target) across all of its retail businesses [89]. Similarly, ShareAction, along with other investors, have called on Morrison’s (another leading UK retailer) to disclose sales-based information and to publish a long-term target and strategy to significantly increase shares of healthier products [90].

Harrington Investments, a small institutional investor that focuses on responsible investing and shareholder advocacy, has also filed several health-related shareholder resolutions at major food companies. This includes a 2019 resolution filed at Coca Cola on ‘Sugar and Public Health’, calling on the company to issue an independent review of its products marketed to consumers, especially those targeted at children, including an assessment of risks to Coca Cola’s finances and reputation. Harington Investments has since filed similar resolutions at PepsiCo and McDonalds. In 2020, 11% of PepsiCo shareholders and 7% (up from 4.9% in 2019) of Coca-Cola’s shareholders voted in favour, which indicates increasing but overall low support for the proposal [91].

## Discussion

This paper has summarized current trends and new developments with regard to institutional investor actions related to nutrition and obesity prevention. We found that nutrition issues appear to be emergent for institutional investors, with evidence that investor interest in nutrition is rising. In particular, material risks related to litigation, regulation and changing consumer preferences are increasingly noted by investors as challenges facing the food industry. The ways in which investors incorporate nutrition within their decision making, and the implications for their research, analysis and engagement varies. Several major investors are publicly scrutinizing the role of food manufacturers and retailers in addressing nutrition issues, and increasing pressure on companies to respond by improving nutrition-related practices. There is also increasing civil society activism focused on investors as a point of leverage in efforts to improve population diets. This builds on activism around other ESG issues, such as those related to climate change and corporate governance [92–94].

There have been a number of recent initiatives that aim to accelerate the transition towards equitable, healthy and sustainable food systems, which focus on leveraging finance to support nutrition goals. In 2021, the UN Food Systems Summit and the Tokyo Nutrition for Growth Summit are two international multi-stakeholder initiatives that may promote further action from the food industry, governments and the financial sector in achieving nutrition-related goals within the SDGs [33, 45]. Recent ESG reporting standards and frameworks developed by groups such as the SASB and the GRI include reporting metrics for nutrition, and are likely to be important for facilitating corporate reporting on nutrition and encouraging the uptake of nutrition-related data by end users including institutional investors [95]. Furthermore, an increasing number of food industry benchmarking and accountability initiatives include nutrition as a key focus area. The Access to Nutrition Initiative, in particular, assesses a wide range of topic areas relevant to nutrition across the life stages, including indicators for undernutrition, overnutrition and infant and children’s nutrition (through their assessment of breast milk substitutes and complimentary foods) [61]. Promisingly, there are also a number of initiatives that take a food systems approach to assessing companies in the food and agricultural sector, including the World Benchmarking Alliance and The Food Foundation’s Plating up Progress initiative which measure corporate practices across nutrition, environment and social inclusion topics [66, 69]. This type of holistic approach to assessing food systems issues will be important for framing complex food issues, such as those related to the expanding alternative proteins market. Importantly, a number of these initiatives target investors as end users and have a specific focus on engagement and translation of findings to investors [59, 60]. Civil society groups and advocacy groups, such as Share Action and The Food Foundation, also play a key role in bringing attention to nutrition issues, particularly in engaging the media to highlight areas in which food companies and investors could do better.

There are, however, a number of issues associated with having multiple ESG standards/reporting frameworks and benchmarking initiatives. Most notably, the relevant initiatives and frameworks currently differ in scope and use different methodologies, thereby risking inconsistent reporting of data by companies, and undermining its use in investment decision-making. There has been some effort to consolidate ESG reporting, with several high profile groups recently committing to collaborate on comprehensive corporate reporting that reduces confusion and overlap [96, 97]. However, the voluntary nature of existing standards and reporting frameworks means that corporate reporting on nutrition, where it exists, is highly variable and not comprehensive [98]. Governments in some jurisdictions, such as the EU, are taking steps to standardise industry and investor reporting on ESG issues through regulation and mandatory disclosure requirements [48], and there is considerable potential to include nutrition-related reporting for relevant companies. Increased methodological alignment between existing benchmarking initiatives will likely facilitate greater food company and investor engagement.

## Conclusions

Comprehensive, wide-spread action from governments, civil society, the food industry and the financial sector is needed to improve the healthiness of population diets. Institutional investors can play a role through influencing the governance and practices of the food industry, whilst helping to hold them accountable for their contribution to unhealthy diets. There is increasing interest from institutional investors in addressing nutrition-related issues; however, investor activity in the area is piece-meal, with large variation in the extent to which issues are considered. There is a need for further integration of nutrition within current reporting standards, alongside comprehensive reporting, monitoring and evaluation of progress on nutrition-related topics by food companies. Methodological alignment across the increasing number of food industry accountability initiatives would likely help galvanise increased investor action in the area. Some jurisdictions are introducing relevant mandatory reporting requirements, which is likely to play a key role in enhancing transparency by the food industry and financial institutions.

## Supplementary Information

Below is the link to the electronic supplementary material.

Supplementary file1 (PDF 278 KB)

## Footnotes

1 Malnutrition is defined by the WHO as undernutrition (wasting, stunting, underweight), inadequate vitamins or minerals, overweight, obesity and resulting diet-related noncommunicable diseases. Source: https://www.who.int/news-room/fact-sheets/detail/malnutrition.

2 The definitions of these 5 areas are: Governance—Core expectation: Defined board management governance processes which routinely review risks from increased regulation of unhealthy food, and sugar in particular; Strategy — Core expectation: Clear strategic goal to adapt to health and wellness trends by providing a balanced portfolio to respond to increasing regulation and changing consumer tastes. Business models assessed for viability in the light of different scenarios for regulation of sugar, promotion of healthy eating; Implementation — Core expectation: Clearly defined plan for achieving strategic goals relating to health and wellness trends. Risk management embedded into processes which ensure business models remain robust in the face of changing policy dynamics; Public Policy Position and Lobbying — Core expectation: Transparency on active engagement with public policy makers at national and international level in a manner consistent with guidance on public health; Demonstrating Progress — Core expectation: Investors are seeking evidence of policy implementation and welcome company views on the most relevant metrics for their sub sector, business model and market focus. Investors also ask for sufficient disclosure or enable a fair comparison of performance between companies.

## References

[1] Branca F, Lartey A, Oenema S, Aguayo V, Stordalen GA, Richardson R, et al. Transforming the food system to fight non-communicable diseases. BMJ. 2019;364:l296. 10.1136/bmj.l296 PMC6349221 30692128

[2] Fanzo J, Haddad L, McLaren R, Marshall Q, Davis C, Herforth A (2020). The Food Systems Dashboard is a new tool to inform better food policy. Nature Food.

[3] Swinburn BA, Kraak VI, Allender S, Atkins VJ, Baker PI, Bogard JR (2019). The global syndemic of obesity, undernutrition, and climate change: the Lancet Commission report. The Lancet.

[4] HLPE. Food security and nutrition: building a global narrative towards 2030. A report by the High Level Panel of Experts on Food Security and Nutrition of the Committee on World Food Security. Rome: HLPE; 2020.

[5] Swinburn B, Dietz W, Kleinert S (2015). A Lancet Commission on obesity. The Lancet.

[6] Swinburn B, Sacks G, Hall K (2011). The global obesity pandemic: shaped by global drivers and local environments. The Lancet.

[7] Popkin BM, Adair LS, Ng SW (2012). Global nutrition transition and the pandemic of obesity in developing countries. Nutr Rev.

[8] Moodie R, Stuckler D, Monteiro C, Sheron N, Neal B, Thamarangsi T (2013). Profits and pandemics: prevention of harmful effects of tobacco, alcohol, and ultra-processed food and drink industries. Lancet (British edition).

[9] Mialon M, Swinburn B, Allender S, Sacks G. Systematic examination of publicly-available information reveals the diverse and extensive corporate political activity of the food industry in Australia. BMC Public Health. 2016;16:283. 10.1186/s12889-016-2955-7 PMC4804618 27004957

[10] Mialon M, Gomes FdS. Public health and the ultra-processed food and drink products industry: corporate political activity of major transnationals in Latin America and the Caribbean. Public Health Nutrition. 2019;22(10):1898–908. 10.1017/S1368980019000417 PMC10260506 30859929

[11] UNEP Finance Initiative. The Principles for Positive Impact Finance: a Common Framework to Finance the Sustainable Development Goals. Geneva: United Nations; 2017.

[12] Schoenmaker Da, Schramade W. Principles of sustainable finance. First edition. ed: Oxford University Press; 2019.

[13] Sandberg J. Towards a theory of sustainable finance: Inquiry Working Paper 15/08. Geneva: United Nations Environment Programme; 2015.

[14] United Nations Environment Programme (UNEP) Finance Initiative. Fiduciary Duty in the 21st Century: Final Report. Geneva: UNEP; 2019.

[15] OECD. The role of Institutional Investors in Promoting Good Corporate Governance. Corporate Governance, OECD Publishing; 2011.

[16] McNulty T, Nordberg D (2016). Ownership, activism and engagement: institutional investors as active owners. Corporate Governance: An International Review.

[17] Gillan SL, Starks LT (2003). Corporate governance, corporate ownership, and the role of institutional investors: a Global Perspective. J Appl Financ.

[18] White M, Aguirre E, Finegood DT, Holmes C, Sacks G, Smith R. What role should the commercial food system play in promoting health through better diet? BMJ. 2020;368:m545. 10.1136/bmj.m545 PMC7190369 32184211

[19] International Panel of Experts on Sustainable Food Systems (IPES-Food). Too big to feed: exploring the impacts of mega-mergers, concentration, concentration of power in the agri-food sector. IPES-Food: Brussels; 2017.

[20] Yach D, Brinchmann S, Bellet S (2001). Healthy investments and investing in health. J Bus Ethics.

[21] Gulland A. Pension funds: tobacco investment up in smoke. BMJ: British Medical Journal. 2016;352(8049):i1491-i. 10.1136/bmj.i1491 26969491

[22] de Bruin B. Socially responsible investment in the alcohol industry: an assessment of investor attitudes and ethical arguments. Contemporary Social Science. 2013.

[23] Hong H, Kacperczyk M (2009). The price of sin: the effects of social norms on markets. J Financ Econ.

[24] Wagemans FAJ, van Koppen CSA, Mol APJ (2013). The effectiveness of socially responsible investment: a review. J Integr Environ Sci.

[25] • Kickbusch I, Krech R, Franz C, Wells N. Banking for health: opportunities in cooperation between banking and health applying innovation from other sectors. BMJ Global Health. 2018;3(Suppl 1):e000598. *This article argues for the explicit inclusion of health ‘H’ criteria within the ESG framework, to create a new ESG+H framework that can be applied to corporate reporting, responsible investment products and responsible investment strategies (e.g., screening).* 10.1136/bmjgh-2017-000598 PMC6001912 29915672

[26] Phillips PJ (2011). Sin stocks in self managed superannuation funds. Australasian Accounting Business & Finance Journal.

[27] Boele N, Bayes S (2020). Responsible investment benchmark report.

[28] • World Economic Forum. Incentivising investment benchmark report. Geneva: World Economic Forum; 2020. *This report outlines a range of actions that stakeholders (e.g.. governments, private sector, civil society, research) can take to facilitate institutional investment pathways that support healthy, sustainable and equitable food systems.*

[29] •• Access to Nutrition Initiative (ATNI). Investor Expectations on Nutrition, Diet and Health. Utrecht: ATNI; 2020. *This report sets out four Investor Expectations related to corporate governance, strategy, lobbying and transparency for food and beverage manufacturers and retailers. Investors that adopt these expectations do so in order to demonstrate their commitment to addressing global nutrition challenges.*

[30] Dourma K, Scott L, Bulzomi A. The SDG Investment Case. The United Nations Principles of Responsible Investment; 2017.

[31] Fanzo J (2019). Healthy and sustainable diets and food systems: the key to achieving Sustainable Development Goal 2?. Food Ethics.

[32] Development Initiatives. Global Nutrition Report 2017: Nourishing the SDG's. Bristol, UK: Development Initiatives; 2017.

[33] Food and Agricultural Organization of the United Nations. United Nations Decade of Action on Nutrition 2016–2015: Mid-term Review Foresight paper. FAO; 2020.

[34] United Nations. Sustainable Development Goals: United Nations; 2015 [8 May 2021]. Available from: http://www.un.org/sustainabledevelopment/sustainable-development-goals/.

[35] Cooper K. Obesity and the SDGs: an opportunity hidden in plain sight: The World Obesity Federation; 2019 [10 June 2021]. Available from: https://www.worldobesity.org/news/blog-obesity-and-the-sdgs-an-opportunity-hidden-in-plain-sight.

[36] United Nations Global Compact (2021). UN Global Compact Strategy 2021–2023.

[37] United Nations Global Compact, KMPG International. SDG Industry Matrix: Financial Services. United Nations; 2015.

[38] United Nations Global Compact, KPMG International. SDG Industry Matrix: Food, Beverage and Consumer Goods. United Nations; 2015.

[39] United Nations Environment Programme Finance Initiative (UNEP FI). About UNEP FI 2021 [2 September 2021]. Available from: https://www.unepfi.org/about/.

[40] United Nations Principles for Responsible Investment. About the PRI: UNPRI; 2021 [2 September 2021]. Available from: https://www.unpri.org/pri/about-the-pri.

[41] United Nations Principles for Responsible Investment. Investor reporting guidance 2021 [18 October 2021]. Available from: https://www.unpri.org/reporting-and-assessment/investor-reporting-guidance/5373.article.

[42] United Nations. The Food Systems Summit 2021 [27 September 2021]. Available from: https://www.un.org/en/food-systems-summit.

[43] Canfield M, Anderson MD, McMichael P. UN Food Systems Summit 2021: Dismantling Democracy and Resetting Corporate Control of Food Systems. Frontiers in Sustainable Food Systems. 2021;5(103).

[44] United Nations. Food Systems Summit 2021: Levers of Change 2021 [27 September 2021]. Available from: https://www.un.org/en/food-systems-summit/levers-of-change.

[45] Tokyo Nutrition For Growth Summit 2021. Vision and Roadmap. Nutrition for Growth; 2021.

[46] Van der Lugt CT, P. P. van de Wijs, & D. Petrovics. Carrots & Sticks 2020 - Sustainability Reporting Policy: Global trends in disclosure as the ESG agenda goes mainstream. Global Reporting Initiative (GRI) and the University of Stellenbosch (USB); 2020.

[47] Heath A, Paty M, Martindale W. Global Guide to Responsible Investment Regulation. United Nations Principles of Responsible Investment (UNPRI); 2016.

[48] Commission E. Directive of the European Parliament and of the Council Amending Directive 2013/34/EU, Directive 2004/109/EC, Directive 2006/43/EC and Regulation (EU) No 537/2014, as regards corporate sustainability reporting. Brussels: European Commission; 2021.

[49] European commission. Regulation (EU) 2019/2088 of the European Parliament and of the Council of 27 November 2019 on sustainability-related disclosures in the financial services sector. Brussels: European Commission; 2019.

[50] Sustainability Accounting Standards Board. Standard Overview 2021 [28 June 2021]. Available from: https://www.sasb.org/.

[51] Global Reporting Initiative. The GRI Standards 2020 [14 Sep 2021]. Available from: https://www.globalreporting.org/how-to-use-the-gri-standards/gri-standards-english-language/.

[52] Global Reporting Initiative (GRI). G4 Sector Disclosures: Food Processing. Amsterdam: Global Reporting Initiative; 2014.

[53] Climate Disclosure Standards Board (CDSB). CDSB Framework for reporting environmental and climate change information. London: CDSB; 2019.

[54] Climate Disclosure Standards Board (CDSB). Corporate reporting on social matters. CDSB position paper. London: CDSB; 2021.

[55] The International Integrated Reporting Council (IIRC). International <IR> Framework. January 2021. IIRC; 2021.

[56] United Nations Global Compact, Global Reporting Initiative. Business Reporting on the SDGs: Analysis of the goals and targets. UN Global Compact, GRI; 2019.

[57] KPMG. The time has come: the KPMG Survey of Sustainability Reporting 2020. KPMG International; 2020.

[58] Schramade W (2017). Investing in the UN Sustainable Development Goals: opportunities for companies and investors. J Appl Corp Financ.

[59] Access to Nutrition Initiative. Access to Nutrition Initiative Investor Statement 2020 [7 July 2021]. Available from: http://accesstonutrition.org/investor-signatories/.

[60] •• The Food Foundation. Plating Up Progress 2021. Section 2c: recommendations for investors. London: Food Foundation; 2021. *The 2021 Food Foundation report puts forward recommendations for ways in which investors can support healthy and sustainable food systems in the UK. This report includes specific ‘asks’ for investors to use in their engagements with businesses and policy makers.*

[61] Access to Nutrition Initiative (2021). Global Index 2021.

[62] Access to Nutrition Initiative (2020). India Spotlight Index 2020.

[63] Access to Nutrition Initiative. U.S. Spotlight Index 2018. Utrecht: ATNI; 2018.

[64] Access to Nutrition Initiative. Investor signatories: ATNI; 2021 [20 October 2021]. Available from: https://accesstonutrition.org/investor-signatories/.

[65] Access to Nutrition Initiative. UK Supermarket Spotlight: a review of the 10 largest UK food retailers' disclosure on nutrition, diets and health. Utrecht; 2020.

[66] World Benchmarking Alliance. Food and Agriculture Benchmark 2021 [2 July 2021]. Available from: https://www.worldbenchmarkingalliance.org/food-and-agriculture-benchmark/.

[67] World Benchmarking Alliance. Food and Agricultural Benchmark 2021 2021 [2 October 2021]. Available from: https://www.worldbenchmarkingalliance.org/publication/food-agriculture/.

[68] Foundation TF, Progress PU (2021). Section 1: analysis of supermarkets, restaurants, caterers and wholesalers.

[69] Nicholson W (2019). Plating up progress: 'must-have' metrics.

[70] Sacks G, Kwon J, Vandevijvere S, Swinburn B (2021). Benchmarking as a public health strategy for creating healthy food environments: an evaluation of the INFORMAS Initiative (2012–2020). Annu Rev Public Health.

[71] INFORMAS. About Us. 2021 [20 Sep 2021]. Available from: https://www.informas.org/about-informas/.

[72] Sacks G, Vanderlee L, Robinson E, Vandevijvere S, Cameron AJ, Ni Mhurchu C, et al. BIA-Obesity (Business Impact Assessment—Obesity and population-level nutrition): A tool and process to assess food company policies and commitments related to obesity prevention and population nutrition at the national level. Obesity Reviews. 2019;0(0). 10.1111/obr.12878 31317645

[73] Sacks G, Robinson E, Cameron AJ, Vanderlee L, Vandevijvere S, Swinburn B. Benchmarking the nutrition-related policies and commitments of major food companies in Australia, 2018. International Journal of Environmental Research and Public Health. 2020;17(17). 10.3390/ijerph17176118 PMC7504100 32842662

[74] Kasture A, Vandevijvere S, Robinson E, Sacks G, Swinburn B (2019). Benchmarking the commitments related to population nutrition and obesity prevention of major food companies in New Zealand. Int J Public Health.

[75] Vanderlee L, Vergeer L, Sacks G, Robinson E, L'Abbé M. Food and beverage manufacturers in Canada: policies and commitments to improve the food environment. Toronto; 2019.

[76] Karupaiah T, Ng SH, Sacks G, Kelly B, Swinburn B, Yeatman H (2019). Benchmarking food industry commitments to create a healthier food environment: business impact assessment (BIA) - Obesity Malaysia 2019.

[77] Vandevijvere S, Van Dam I. Food companies' commitments and practices on food environments and population nutrition in Belgium: a detailed assessment. Company assessments and recommendations using the 'Business Impact Assessment on Οbesity and Population Nutrition' (BIA-Obesity). Belgium. 2021.

[78] Vandevijvere S, Dam IV. Food companies’ commitments and practices on food environments and nutrition in Belgium: a detailed assessment. Company assessments and recommendations using the Business Impact Assessment on obesity and population nutrition (BIA-Obesity). Brussels: Sciensano; 2021.

[79] Russell F (2017). FTSE4Good inclusion criteria for the marketing of breast milk substitutes.

[80] •• Robinson E, Parker C, Carey R, Sacks G. The extent to which obesity and population nutrition are considered by institutional investors engaged in responsible investment in Australia - a review of policies and commitments. 2020;11(3647). *This article explores the extent to which a leading practice sample of institutional investors in australia report on their approach to considering nutrition and obesity-related issues.* 10.3389/fpsyg.2020.577816 PMC7793752 33424688

[81] Sacks G, Robinson E (2018). Investing for health: potential mechanisms for the investment community to contribute to obesity prevention and improved nutrition. Curr Obes Rep.

[82] •• Irving E, Crossman M. Sugar, obesity and noncommunicable diseases: investor expectations. London: Schroders and Rathbone Greenbank Investments; 2017. *This report outlines five core areas where investors would welcome greater transparency from companies that derive revenue from the sale of food and beverage products or ingredients, and explains how investors may use this information. The report also suggests KPIs for increased transparency from companies.*

[83] Credit Suisse Research Institute. Sugar: Consumption at a crossroads. Credit Suisse;2013.

[84] Witte B, Obloj P, Koktenturk S, Morach B, Brigl M, Rogg J, et al. Food for thought: the protein transformation. BCG, Blue Horizon; 2021.

[85] Kraak VI. Perspective: unpacking the wicked challenges for alternative proteins in the United States: can highly processed plant-based and cell-cultured food and beverage products support healthy and sustainable diets and food systems? Advances in Nutrition. 2021. 10.1093/advances/nmab113 PMC8803483 34662900

[86] Rosewarne E, Farrand C. Salt levels in meat alternatives in Australia (2010–2019). The George Institute for Global Health; 2019.

[87] Lacy-Nichols J, Hattersley L, Scrinis G (2021). Nutritional marketing of plant-based meat-analogue products: an exploratory study of front-of-pack and website claims in the USA. Public Health Nutr.

[88] Rathbone Greenbank Investments. Shareholders urge action from UK government following the National Food Strategy’s recommendations 2021 [16 Sep 2021]. Available from: https://www.rathbonegreenbank.com/insight/shareholders-urge-action-uk-government-following-national-food-strategys-recommendations.

[89] • ShareAction. Tesco makes further health commitments in response to investor engagement. ShareAction; 2021 [16 Sep 2021]. Available from: https://shareaction.org/tesco-makes-further-health-commitments-in-response-to-investor-engagement/. *This is a recent high profile example of shareholder advocacy at a major food retailer in the UK, which reportedly led the company to strengthen their nutrition-related policies and reporting practices.*

[90] ShareAction. $1.1tn investors call on Morrisons to boost sales of healthy products: ShareAction; 2021 [16 Sep 2021]. Available from: https://shareaction.org/1-1tn-investors-call-on-morrisons-to-boost-sales-of-healthy-products/.

[91] FoodDive. Coca-Cola and PepsiCo proxy clash with activist investor over sugar hints at future skirmishes. 2021 [16 Sep 2021]. Available from: https://www.fooddive.com/news/coca-cola-and-pepsico-proxy-clash-with-activist-investor-over-sugar-hints-a/598146/.

[92] Guay T, Doh JP, Sinclair G (2004). Non-governmental organizations, shareholder activism, and socially responsible investments: ethical, strategic, and governance implications. J Bus Ethics.

[93] Michael M, Jacob P (2011). Financial activism and global climate change: the rise of investor-driven governance networks.

[94] Foerster A, Sheehan K, Parris D. Investing for a safe climate? University of NSW Law Journal. 2021;44(4).

[95] Wellesley L, Eis J, Marijs C, Vexler C, Waites F, Benton TG (2020). Chatham House Report: the Business Case for Investment in Nutrition.

[96] CDP, CDSB, GRI, IIRC and SASB,. Statement of Intent to Work Together Towards Comprehensive Corporate Reporting: Summary of alignment discussions among leading sustainability and integrated reporting organisations CDP, CDSB, GRI, IIRC and SASB. Climate Disclosure Project (CDP), Climate Disclosure Standards Board (CDSB), Global Reporting Initiative (GRI), International Integrated Reporting Council (IIRC), Sustainability Accounting Standards Board (SASB); 2020.

[97] World Economic Forum (2020). Toward Common Metrics and Consistent Reporting of Sustainable Value Creation.

[98] KPMG International. The Road Ahead: The KPMG Survey of Corporate Responsibility Reporting 2017. The Netherlands; 2017.

