# Supplementary material for: Latest Trends in Investing for Improved Nutrition and Obesity Prevention
Source: Curr Nutr Rep. 2022 Jan 26;11(1):39–55. doi: 10.1007/s13668-021-00389-7 (PMC8942889; doi:10.1007/s13668-021-00389-7)
Supplement: Supplementary file 1 — Supplementary file1 (PDF 278 KB) [file 13668_2021_389_MOESM1_ESM.pdf]

Supplementary file Table S1.

Title: Latest trends in investing for improved nutrition and obesity prevention

Ella Robinson[1], Rachel Carey[2], Anita Foerster[3] and Gary Sacks[1]*

Contact information

Ella Robinson* ella.robinson@deakin.edu.au
Rachel Carey rachel.carey@unimelb.edu.au
Anita Foerster anita.foerster@monash.edu
Gary Sacks gary.sacks@deakin.edu.au
*Corresponding author

Affiliations

[1]Deakin University, Geelong, Institute for Health Transformation, Global Obesity Centre (GLOBE), VIC, Australia.

[2]The University of Melbourne, Melbourne, School of Agriculture and Food, VIC, Australia.
[3]Monash University, Melbourne, Monash Business School, VIC, Australia.

Table S1: Indicators and metrics across non-governmental Environmental Social Governance (ESG) reporting standards and frameworks that include relevant 'social' or nutrition-related topics[1]

Organisation	Standard/framework	Description	User	Audience	Nutrition-related topics, indicators and metrics
**The Global Reporting Initiative (GRI)**	GRI Standards (55)	The GRI Standards are one of the most prominent and commonly used sustainability reporting frameworks, providing standards for companies to report on environmental, social and economic impacts. The GRI Standards include a set of universal standards and topic-specific standards that are selected by the company based on materiality.	Any organization	Companies, investors, policymakers, capital markets, and civil society	GRI 401-419 includes 19 'social' topic specific reporting standards. Reporting requirements under each standard that may be relevant to nutrition include:<br><br>**GRI 414: Supplier social assessment**<br>Reporting requirements<br>414 -1: New suppliers that were screened using social criteria<br>The reporting organization shall report the following information:<br>a. Percentage of new suppliers that were screened using social criteria.<br><br>414 -2: Negative social impacts in the supply chain and actions taken<br>The reporting organization shall report the following information:<br>a. Number of suppliers assessed for social impacts.<br>b. Number of suppliers identified as having significant actual and potential negative social impacts.<br>c. Significant actual and potential negative social impacts identified in the supply chain.<br>d. Percentage of suppliers identified as having significant actual and potential negative social impacts with which improvements were agreed upon as a result of assessment.<br>e. Percentage of suppliers identified as having significant actual and potential negative social impacts with which relationships were terminated as a result of assessment, and why.<br><br>**GRI 415: Public policy**<br>Reporting requirements<br>415-1: Political contributions<br>The reporting organization shall report the following information:<br>a. Total monetary value of financial and in-kind political contributions made directly and indirectly by the organization by country and recipient/beneficiary.<br>b. If applicable, how the monetary value of in-kind contributions was estimated.Total monetary value of financial and in-kind political contributions made directly and indirectly by the organization by country and recipient/beneficiary.<br><br>**GRI 417: Marketing and labelling**<br>Reporting requirements<br>417-1: Requirements for product and service information and labeling

					The reporting organization shall report the following information:<br>a. Whether each of the following types of information is required by the organization’s procedures for product and service information and labeling:<br>i. The sourcing of components of the product or service;<br>ii. Content, particularly with regard to substances that might produce an environmental or social impact;<br>iii. Safe use of the product or service;<br>iv. Disposal of the product and environmental or social impacts;<br>v. Other (explain).<br>b. Percentage of significant product or service categories covered by and assessed for compliance with such procedures.<br><br>417-2: Incidents of non-compliance concerning product and service information and labeling<br>The reporting organization shall report the following information:<br>a. Total number of incidents of non-compliance with regulations and/or voluntary codes concerning product and service information and labeling, by:<br>i. incidents of non-compliance with regulations resulting in a fine or penalty;<br>ii. incidents of non-compliance with regulations resulting in a warning;<br>iii. incidents of non-compliance with voluntary codes.<br>b. If the organization has not identified any non-compliance with regulations and/or voluntary codes, a brief statement of this fact is sufficient.<br><br>417-3: Incidents of non-compliance concerning marketing communications<br>The reporting organization shall report the following information:<br>a. Total number of incidents of non-compliance with regulations and/or voluntary codes concerning marketing communications, including advertising, promotion, and sponsorship, by:<br>i. incidents of non-compliance with regulations resulting in a fine or penalty;<br>ii. incidents of non-compliance with regulations resulting in a warning;<br>iii. incidents of non-compliance with voluntary codes.<br>b. If the organization has not identified any non-compliance with regulations and/or voluntary codes, a brief statement of this fact is sufficient.
	G4 Sector Disclosures (superseded by GRI Standards) (56)	The GRI previously developed the G4 Sector Disclosures, which provided sector-specific reporting guidelines. The G4 Sector	Airport Operators, Construction and Real Estate, Electric Utilities, Event Organizers,	Companies, investors, policymakers, capital	**Topic: Society - Public Policy**<br>**Guidance:** Food processing companies should provide clarity and specific detail on any lobbying activities related to the subsidized or otherwise advantaged production of key product ingredients within their organization.

		Disclosures were superseded by the GRI Standards and are not required for preparing a report in accordance with the GRI Standards, however can still be used to provide additional sector-specific guidance [2].	Financial Services, **Food Processing**, Media, Mining and Metals, NGO, Oil and Gas	markets, and civil society	**Topic: Society - Healthy and Affordable Food**<br>**Guidance:** Nature, scope and effectiveness of any programs and practices (in-kind contributions, volunteer initiatives, knowledge transfer, partnerships and product development) that promote access to healthy lifestyles; the prevention of chronic disease; access to healthy, nutritious and affordable food; and improved welfare for communities in need.<br><br>**Topic: Product Responsibility - Customer Health and Safety**<br>**Indicators:**<br>• Indicator FP6: Percentage of total sales volume of consumer products, by product category, that are lowered in saturated fat, trans fats, Sodium and added sugars<br>• Indicator FP7: Percentage of total sales volume of consumer products, by product category, that contain increased nutritious ingredients like fiber, Vitamins, minerals, phytochemicals or functional food additives.<br><br>**Topic: Product Responsibility - Product and Service Labelling.**<br>**Guidance:** Policies and practices on communication to consumers about ingredients and nutritional information beyond legal requirements.<br><br>**Topic: Product Responsibility - Marketing communications.**<br>**Guidance:** When reporting any codes or voluntary standards relating to marketing communications, consider e.g., television, internet, text messages, email, in-school promotions, competitions and giveaways. Food processing companies should also make specific reference to policies and guidelines relating to marketing to vulnerable groups such as expectant and new mothers, children, teenagers and disadvantaged people.
**Sustainability Accounting Standards Board (SASB)[3]**	**SASB Standards (51)**	The Sustainability Accounting Standards Board (SASB) provides sustainability accounting standards for companies to disclose financially material environmental, social and governance information to investors. Includes qualitative and quantitative metrics to measure performance across ESG topics.	Consumer Goods, Extractives and Minerals Processing, Financials, **Food and Beverage[4]**, Health Care, Infrastructure, Renewable Resources and Alternative Energy, Resource Transformation, Services, Technology and	Investors	**Industry: Restaurants**<br>**Topic: Nutritional Content**<br>**Indicators:**<br>• FB-RN-260a.1. (1) Percentage of meal options consistent with national dietary guidelines and (2) revenue from these options<br>• FB-RN-260a.2. (1) Percentage of children's meal options consistent with national dietary guidelines for children and (2) revenue from these options<br>• FB-RN-260a.3. Number of advertising impressions made on children, percentage promoting products that meet national dietary guidelines for children<br><br>**Industry: Processed Foods**<br>**Topic: Health and Nutrition**<br>**Indicators:**

			Communications, Transportation		• FB-PF-260a.1. Revenue from products labeled and/or marketed to promote health and nutrition attributes<br>• FB-PF-260a.2. Discussion of the process to identify and manage products and ingredients related to nutritional and health concerns among consumers<br>**Topic: Product Labelling and Marketing**<br>**Indicators:**<br>• FB-PF-270a.1. Percentage of advertising impressions (1) made on children and (2) made on children promoting products that meet dietary guidelines<br>• FB-PF-270a.2. Revenue from products labeled as (1) containing genetically modified organisms (GMOs) and (2) non-GMO<br>• FB-PF-270a.3. Number of incidents of non-compliance with industry or regulatory labeling and/or marketing codes<br>• FB-PF-270a.4. Total amount of monetary losses as a result of legal proceedings associated with labeling and/or marketing practices<br><br>**<u>Industry: Non-alcoholic beverages</u>**<br>**Topic: Health and Nutrition**<br>**Indicators:**<br>• FB-NB-260a.1. Revenue from (1) zero- and low-calorie, (2) no-added-sugar, and (3) artificially sweetened beverages<br>• FB-NB-260a.2. Discussion of the process to identify and manage products and ingredients related to nutritional and health concerns among consumers<br>**Topic: Product Labelling and Marketing**<br>**Indicators:**<br>• FB-NB-270a.1. Percentage of advertising impressions (1) made on children and (2) made on children promoting products that meet dietary guidelines<br>• FB-NB-270a.2. Revenue from products labeled as (1) containing genetically modified organisms (GMOs) and (2) non-GMO<br>• FB-NB-270a.3. Number of incidents of non-compliance with industry or regulatory labeling and/or marketing codes<br>• FB-NB-270a.4. Total amount of monetary losses as a result of legal proceedings associated with marketing and/or labeling practices<br><br>**<u>Industry: Food Retailers and Distributors</u>**<br>**Topic: Product Health and Nutrition**<br>**Indicators:**<br>• FB-FR-260a.1. Revenue from products labeled and/or marketed to promote health and nutrition attributes<br>• FB-FR-260a.2. Discussion of the process to identify and manage products and ingredients related to nutritional and health concerns among consumers<br>**Topic: Product Labelling and Marketing**

					**Indicators:**<br>• FB-FR-270a.1. Number of incidents of non-compliance with industry or regulatory labeling and/or marketing codes<br>• FB-FR-270a.2. Total amount of monetary losses as a result of legal proceedings associated with marketing and/or labeling practices<br>• FB-FR-270a.3. Revenue from products labeled as (1) containing genetically modified organisms (GMOs) and (2) non-GMO
**Global Reporting Initiative and United Nations Global Compact**	**Business Reporting on the Sustainable Development Goals (SDGs)**	Business Reporting on the SDGs' is an initiative aimed at companies that leverage the GRI Standards and the Ten Principles of the UN Global Compact to embed the SDGs within existing business and reporting processes. The initiative publishes guidelines for companies to integrate SDGs within corporate reporting.	All businesses, regardless of size, sector or operating location	Shareholders and other stakeholders: governments, civil society, consumers and academia	**SDG2: Zero Hunger**<br>**Target 2.1: By 2030, end hunger and ensure access by all people, in particular the poor and people in vulnerable situations, including infants, to safe, nutritious and sufficient food all year round.**<br><br>***Possible relevant business actions* to help achieve this target:***<br>• Respecting human rights and recognizing the violations of human rights that underlie hunger and malnutrition, including poverty, deprivation of land rights, and discrimination. Respecting the universal right to an adequate standard of living, including access to safe and nutritious food. Recognizing businesses' own influence on hunger and people's access to food, particularly the poor and people in vulnerable situations, such as smallholder farmers and agricultural workers (particularly women) who produce much of the world's food but often live in conditions of poverty.<br>• Improving production, conservation and distribution of food by making full use of (existing and new) technical and scientific knowledge, by educating the public on the principles of nutrition and by partnering with other stakeholders to develop or reform agrarian systems to better utilize natural resources.<br>• Improving the availability of nutritious food through product development, relative pricing and providing responsible communications, such as nutritional information, storage and safe use.<br><br>**Target 2.2: By 2030, end all forms of malnutrition, including achieving, by 2025, the internationally agreed targets on stunting and wasting in children under 5 years of age, and address the nutritional needs of adolescent girls, pregnant and lactating women and older persons**<br><br>***Possible relevant business actions* to help achieve this target:***<br>• Recognizing businesses' significant influence on people's diets and access to food. Providing food that contributes to a healthy and balanced diet. Pricing nutritious food options fairly to enable people to afford it considering their purchasing power.<br>• Providing sufficient information about products, including nutrition information, to enable customers to make informed choices.

					• Raising the awareness of employees on health issues including nutrition through training, counselling and other workplace programs.<br><br><u>SDG3: Good Health and Wellbeing</u><br>**Target 3.3: By 2030, end preventable deaths of newborns and children under 5 years of age, with all countries aiming to reduce neonatal mortality to at least as low as 12 per 1,000 live births and under-five mortality to at least as low as 25 per 1,000 live births.**<br><br>***Possible relevant business actions* to help achieve this target:***<br>• Providing access to health-care for employees and their families and encouraging access to health-care for those in the supply chain. Encouraging healthy lifestyles and providing decent working conditions which enable parents to complete their roles as caregivers, such as providing health-care, on-site vaccinations and health screening programs, paying at the minimum the living wage, providing maternity and paternity leave, developing appropriate family-friendly accommodations for workers, and providing facilities necessary for early years care (such as breast feeding rooms, child care facilities and flexible working hours) and access to affordable nutritious food for mothers in the workplace. Implementing suitable risk procedures for expectant mothers to protect them from potential harm to themselves or the fetus in the workplace.<br>• Combatting disease and malnutrition by providing adequate nutritious foods and clean drinking-water, taking into consideration the dangers and risks of environmental pollution. Developing preventive health care guidance for parents and family planning education and services as part of the businesses’ health care routine.<br><br>**Target 3.4: By 2030, reduce by one third premature mortality from non-communicable diseases through prevention and treatment and promote mental health and well-being**<br><br>***Possible relevant business actions* to help achieve this target:***<br>• Supporting access to preventative health care, including through medical coverage offered to employees and their families and company programs focused on health and well-being (e. g. exercise and health programs, smoking cessation programs, weight management and mental health helplines).<br>• Taking responsibility to protect consumers and end-users from any potentially negative health impacts from ingredients, products, services and marketing activities. Working proactively to minimize any known negative and improve any known positive health impacts. Informing the public about

					any risks that an ingredient, product or service may entail for the short and long-term health of the users, and working towards innovations that provide affordable medicines.<br>• Supporting governmental efforts to reduce non-communicable diseases, for example regarding the use of sugar, salt and fat in food production.
**Climate Disclosure Standards Board (CDSP)**	**CDSB Framework for reporting environmental and climate change information (57)**	The CDSP offers a framework for corporate reporting of environmental information in mainstream reports, that aligns with and complements the objective of financial reporting (using metrics and KPIs developed by other standards organizations like CDP, GRI, SASB). It allows investors to assess the relationship between specific environmental matters and the organization's strategy, performance and prospects.	Organisations, including single companies or entities and corporate groups	Investors	The CDSP is working to expand the scope of its reporting framework to include financially material social issues, with the aim of releasing the new framework in 2021 (58). It is not clear whether nutrition will be included within the scope of this new framework.
**The International Integrated Reporting Council (IIRC)[3]**	**International Integrated Reporting (<IR>) Framework (59)**	The purpose of the <IR> Framework is to establish principles and elements of an integrated report, which is a concise communication about how an organization's strategy, governance, performance and prospects, lead to the creation, preservation or erosion of value over the short, medium and long term.	Private sector, for-profit companies of any size. Can also be applied, adapted as necessary, by public sector and not-for-profit organizations	Providers of financial capital	The IR Framework defines various forms of capital (resources and relationships used and affected by the organization), including financial, manufactured, intellectual, human, social and relationship, and natural. Company activities related to nutrition may fit under social and relationship capital.<br><br>**Social and relationship** capital includes:<br>The institutions and the relationships within and between communities, groups of stakeholders and other networks, and the ability to share information to enhance individual and collective well-being.<br>Nutrition fits in under:<br>– Shared norms, and common values and behaviours<br>– Key stakeholder relationships, and the trust and willingness to engage that an organization has developed and strives to build and protect with external stakeholders<br>– Intangibles associated with the brand and reputation that an organization has developed<br>– An organization's social licence to operate.

[1] ESG reporting standards and frameworks that only include topics related to environment/climate change are not included in this table. The most prominent of these are the Taskforce on Climate related Financial Disclosures (TCFD) and the Climate Disclosure Project (CDP).
[2] The GRI is working on new 'Sector Standards' which will describe a sector's most significant impacts from a sustainable development perspective. As at 2021, standards have been developed for the 'Oil and Gas Sector' and 'Agriculture, Aquaculture and Fishing Sector'.
[3] In November 2020 the International Integrated Reporting Council (IIRC) and the Sustainability Accounting Standards Board (SASB) announced their intention to merge into the Value Reporting Foundation, which was officially formed in June 2021. The SASB Standards are now maintained under the auspices of the Value Reporting Foundation.
[4] Food and beverage includes 8 industries: Food retailers and distributers, Meat, poultry and dairy, Alcoholic beverages, Non-alcoholic beverages, Processed foods, Restaurants, Tobacco and Agricultural products.
[*] Only nutrition and nutrition-related non-communicable disease relevant business actions/opportunities were extracted and reported.
